# Distal Humeral Trochlear Geometry Associated With the Spatial Variation of the Dynamic Elbow Flexion Axis

**DOI:** 10.3389/fbioe.2022.850198

**Published:** 2022-06-24

**Authors:** Diyang Zou, Xiangjun Hu, Kai-Nan An, Kerong Dai, Xiaowei Yu, Weihua Gong, Tsung-Yuan Tsai

**Affiliations:** ^1^ School of Biomedical Engineering & Med-X Research Institute, Shanghai Jiao Tong University, Shanghai, China; ^2^ Department of Orthopaedic Surgery, Shanghai Ninth People s Hospital, Shanghai Key Laboratory of Orthopaedic Implants & Clinical Translation R&D Center of 3D Printing Technology, Shanghai Jiao Tong University School of Medicine, Shanghai, China; ^3^ Department of Biomechanics, Mayo Clinic, Rochester, MN, United States; ^4^ Department of Orthopaedic Surgery, Shanghai Jiao Tong University Affiliated Sixth People’s Hospital, Shanghai, China; ^5^ Department of Rehabilitation Medicine, Zhongshan Hospital, Fudan University, Shanghai, China; ^6^ Engineering Research Center of Digital Medicine and Clinical Translation, Ministry of Education, Shanghai, China

**Keywords:** humeroulnar joint, elbow flexion axis, flexion–extension movement, morphology, distal humeral trochlea

## Abstract

**Background:** The complexity of the spatial dynamic flexion axis (DFA) of the elbow joint makes the elbow prosthesis design and humeral component alignment challenging. This study aimed to 1) investigate the variations of the spatial DFA during elbow flexion and 2) investigate the relationship between the distal humeral trochlear geometry and the *in vivo* spatial variation of the DFA.

**Methods:** Ten healthy subjects participated in this study. Each subject performed a full elbow extension to maximum flexion with hand supination under dual fluoroscopic imaging system (DFIS) surveillance. The 2D fluoroscopic images and the 3D bone models were registered to analyze the *in vivo* elbow kinematics and DFAs. The spatial DFA positions were defined as inclination with the medial and lateral epicondyle axes (MLA) in the transverse and coronal planes. The range of the DFA positions was also investigated during different flexion phases. The Spearman correlation method was used to analyze the relationship between the distal humeral trochlear’s morphological parameters and the position of DFAs during different flexion phases.

**Results:** The pathway of the DFAs showed an irregular pattern and presented individual features. The medial trochlear depth (MTD) (*r* = 0.68, *p* = 0.03) was positively correlated with the range of the DFA position (2.8° ± 1.9°) in the coronal plane from full extension to 30° of flexion. Lateral trochlear height (LTH) (*r* = −0.64, *p* = 0.04) was negatively correlated with the DFA position (−1.4° ± 3.3°) in the transverse plane from 30° to 60° of flexion. A significant correlation was found between LTH with the DFA position in the coronal (*r* = −0.77, *p* = 0.01) and transverse planes (*r* = −0.76, *p* = 0.01) from 60° to 90° of flexion.

**Conclusion:** This study showed that the pathway of the dynamic flexion axis has an individual pattern. The medial and lateral trochlear sizes were the key parameters that might affect the elbow joint flexion function. When recovering complex distal humeral fractures or considering the implant design of total elbow arthroplasty, surgeons should pay more attention to the medial and lateral trochlea’s geometry, which may help restore normal elbow kinematics.

## Introduction

Total elbow arthroplasty (TEA) is an effective treatment to replace damaged elbow articular surfaces for patients with rheumatoid arthritis, osteoarthritis, serious distal humeral fracture, or bone tumors (Sanchez-Sotelo, 2011; Mansat et al., 2013b; Krukhaug et al., 2018). Although TEA can relieve the symptoms and restore the elbow’s function for the patient, many postoperative complications such as aseptic loosening, infection, postoperative instability, and periprosthetic fractures were recorded (Schoch et al., 2017; Pham et al., 2018; Geurts et al., 2019). Recent clinical studies have reported a high revision rate of 25% and a high complication rate of 27%–43% after TEA within 10 years (Mansat et al., 2013a; Schoch et al., 2017; Pham et al., 2018; Geurts et al., 2019). Several studies have suggested that the elbow flexion axis mismatch during TEA surgery may lead to high complications (Brownhill et al., 2012). Therefore, it is crucial to determine the axis position accurately when performing TEA surgery.

Accurate identification of the elbow flexion axis during the TEA surgery remains challenging. In clinical practices, the trochlea central axis (TCA) is usually considered as the anatomical elbow flexion axis (Brownhill et al., 2006; McDonald et al., 2010; Wiggers et al., 2014). Traditionally, the position of the TCA is well identified by using lateral X-ray imaging. Surgeons aim to overlap the capitalism and the trochlear contour until they create concentric circles with their centers representing the elbow rotation axis (Wiggers et al., 2017). However, the anatomical bow of the distal humerus and the limb’s position may cause an imperfect lateral X-ray projection and errors in identifying the flexion axis (Dos Santos et al., 2017). Previous studies have demonstrated that these errors could reach up to 10° in the coronal and transverse planes (Brownhill et al., 2006; Wiggers et al., 2014). The mean flexion axis (MFA), known as the functional rotation axis, was another conception for the description of the joint movement (Ehrig et al., 2007; Gordon and Dapena, 2013). The humeral side was fixed while the forearm was flexed freely so that the MFA of the elbow joint was calculated using a motion capture system (Stokdijk et al., 1999; Song et al., 2018). Some researchers recommended MFA as a more appropriate way for rotation axis alignment than the intraoperative X-ray imaging method (Song et al., 2018). However, most MFA calculations rely on image-based navigation or motion capture systems, which are not available in every hospital. Besides, there is no consensus on which referencing method is the best for determining the elbow flexion axis for TEA.

The helical axis theory proposed by Woltring et al. (1985) was commonly used to calculate the dynamic flexion axis (DFA) of joints from acquired kinematics data in biomechanical or clinical studies (Song et al., 2018; Ehrig and Heller, 2019). Previous studies have reported that the DFA during elbow flexion was not fixed but moved like a twist around an axis (Bottlang et al., 2000; Goto et al., 2004). Most of the studies about DFA were based on cadaveric specimens (Duck et al., 2003a; Duck et al., 2003b; Muriuki et al., 2012). It cannot reproduce the flexion motion without muscle activation. The *in vivo* continuous flexion motion measurement plays an essential role in understanding the elbow flexion movement pattern. Studies have shown that various factors would affect elbow motion, including the integrity of the ligament, the employing load, the effect of forearm rotation (pronation or supination), the active or passive motions, and geometrical characters (Duck et al., 2003a; Duck et al., 2003b; Hua et al., 2020). Morphologic studies about the distal elbow showed that the articular shape might vary among people, including the capitellum or trochlear diameters, articular width, and anatomic bow (Brownhill et al., 2007; Desai et al., 2014; Lenoir et al., 2015a). However, the current elbow prosthesis design does not reproduce the complicated distal humeral anatomical structure. This could be another reason which leads to a higher failure rate of TEA (Geurts et al., 2019). The relationship between distal humeral anatomical features with a spatial DFA position is still unclear. Understanding the characteristics of DFA can provide an insight into the elbow prosthesis design and help surgeons determine which anatomic parameters are critical when reconstructing a severely damaged distal humeral trochlear.

The purposes of the present study were to 1) investigate the variations of DFA during *in vivo* elbow flexion–extension movement, and 2) investigate the relationship between the anatomical parameters of the distal humerus with the variation of the DFA in different ranges of flexion. We hypothesized that the DFA of each subject might have an individual movement pattern. The individual height and diameter of the trochlear might correlate with the position of the DFA.

## Materials and Methods

### Subjects and Image Protocol

Ten healthy subjects were recruited in this study (six men and four women, with 10 right elbows). The study was approved by the Institution’s Review Board, and all subjects signed and informed consent before participating in the experiment. The average age was 21.1 ± 0.8 years. The average height and body weights were 171.9 ± 5.4 cm and 65.2 ± 14.6 kg, respectively, and the average BMI was 21.8 ± 4.1. Each subject filled out the American Shoulder and Elbow Surgeons Shoulder Score (ASES) questionnaires with 100 points scored. The dominant hand of all subjects was the right. All subjects included in this research had no history of elbow injury or other musculoskeletal and neural diseases.

**FIGURE 1:**
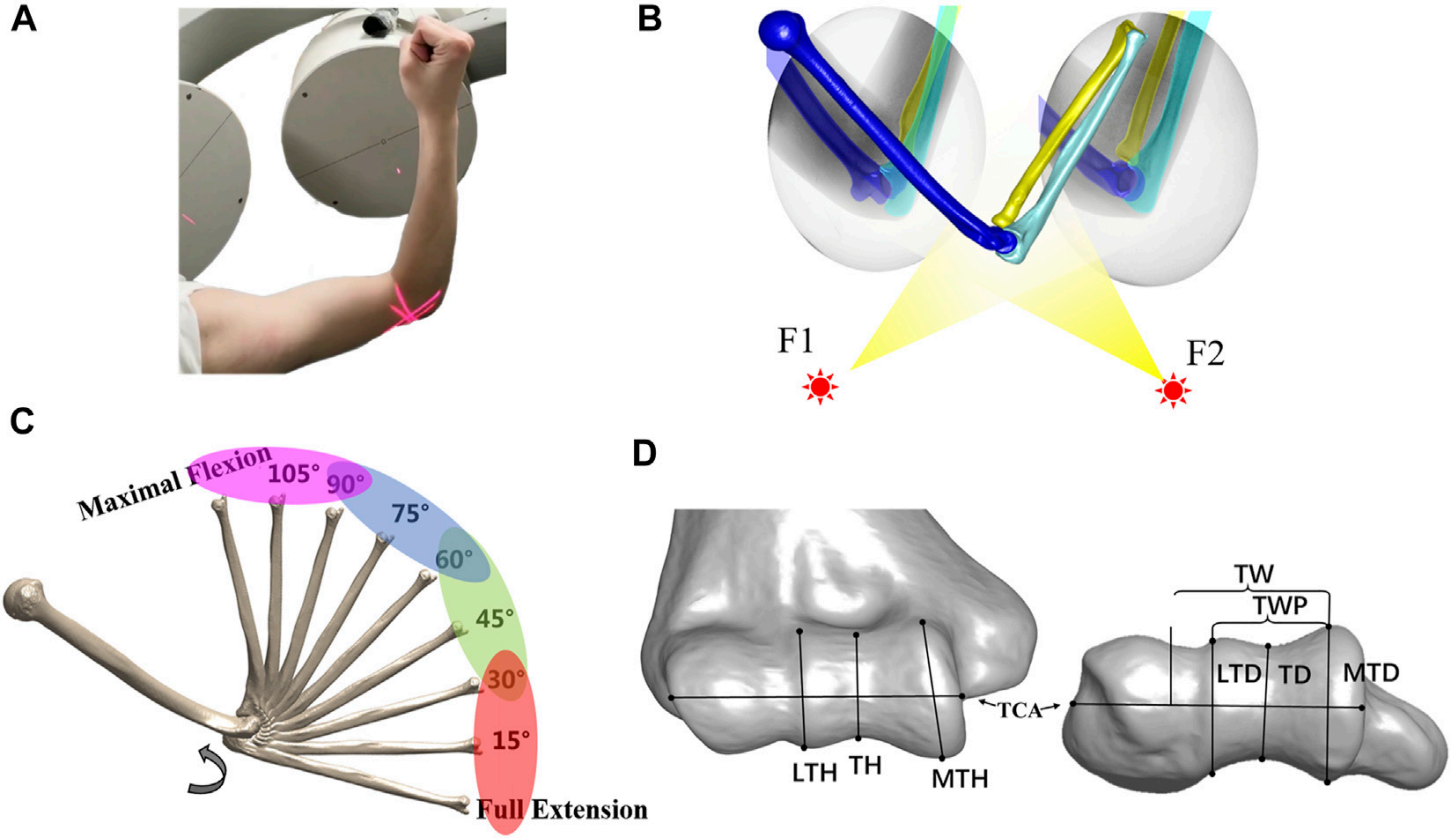
Experimental workflow. **(A)** Each subject performed a full extension to maximal flexion under a biplane fluoroscopic imaging system. **(B)** Virtual experiment environment of 3D–2D registration for acquiring the *in vivo* elbow flexion kinematics. **(C)** Range of the flexion was divided into four phases at full extension to 30°, 30°–60°, 60°–90°, and 90° to maximal flexion. The helical axis theory was applied to calculate the dynamic flexion axis (DFA) at each phase. **(D)** Measurement of distal humeral trochlea morphology. Lateral trochlear height (LTH), trochlear height (TH), and medial trochlear height (MTH) in the coronal plane. Trochlear width (TW), trochlear width proper (TWP), lateral trochlear depth (LTD), trochlear groove depth (TD), and medial trochlear depth (MTD) in the transverse plane.

All subjects received computed tomography (CT) scans (SOMATOM Definition AS, Siemens, Germany) from the wrist to the humeral head. We set the scan voltage and current to 100 KV and 65 mA with an image resolution of 512 × 512 pixels and a voxel size of 0.4 mm^3^ × 0.4 mm^3^ × 0.6 mm^3^. A software platform for three-dimensional (3D) data visualization, Amira (v.6.7.0, Thermo Fisher Scientific, Waltham, MA, United States), was used to generate the 3D model of the humerus, ulna, and radius from the CT images. All subjects completed a full extension to maximum flexion elbow movement with hand supination under a dual fluoroscopic imaging system (DFIS) (BV Pulsera, Philips, Dutch) at a 30 Hz sampling rate. The average time from extension to flexion was 2 s. Two static images of the elbow at full extension and maximum flexion positions with hand supination were also captured for all subjects to calculate the elbow’s range of motion (Figure 1A).

### Registration Procedure

After the experiment, a series of fluoroscopic images were imported into a custom modeling software application MATLAB (MATLAB, MathWorks, Natick, MA, United States). The CT-based bone 3D surface model was introduced into the software for translation and rotation until the projection of the surface model matched the contour of the bone captured on the selected perspective image. The process was repeated every 0.1 s (every three frames) from the maximum straightening position (0 s) to the maximum flexion position (2 s) to complete the registration of the entire moving process (Figure 1B).

### Definition of the Elbow Coordinate System

To describe the spatial movement of elbow flexion in clinically relevant terms, we defined the local anatomic coordinate system of the humerus and ulna using three orthogonal vectors (Wu et al., 2005). The origin of the humeral coordinate system was determined at the medial point of the humeral medial–lateral epicondyle axis (MLA). The medial and lateral epicondyles were located by finding the center of their bony protrusion. The *Y-axis* was from the origin to the center of the best fit sphere of the humeral head, indicating the proximal-distal (PD) direction. The *X-axis* was defined as the vector cross product of the PD-axis and medial–lateral epicondyle axis, showing the anterior–posterior (AP) direction. The *Z-axis* was defined as the cross product of *Y-axis* and *Z-axis*, indicating the medial–lateral (ML) direction. The coordinate system of the ulna was defined with the origin of the ulnar notch center. The *Y-axis* pointed proximally from the distal ulnar styloid to the origin. The *X-axis* was defined as the line perpendicular to the plane formed by the ulnar styloid, humeral lateral epicondyle, and humeral medial epicondyle, pointing to the front. The *Z-axis* was defined as the cross product of the *Y-axis* and *Z-axis*, pointing laterally. The six-degree-of-freedom (6-DOF) kinematics of the elbow was calculated using the Euler angle with a *Z-X-Y* sequence based on the registered bone positions. The average errors of this technique applied in elbow kinematics evaluation were less than ±1.0 mm and ±1.0° (McDonald et al., 2012).

### Rotation Axis Calculation

We used a Gaussian filter to smooth kinematic data by assuming that the noise follows the Gaussian distribution. First, the original transformation matrix was converted to the 6-DOF kinematic data. Second, the 6-DOF kinematic data were smoothed by using a Gaussian filter (The window width of the Gaussian filter was 10 points). The filter parameters were optimized by minimizing the difference between the smoothed data and the raw kinematic data. Finally, the smoothed kinematic data were converted to transformation matrices for calculating the DFA for each subject. *In vivo* kinematics was measured at every interval of 1*°* from full extension to maximal elbow flexion for the comparison of the results with those presented in the previous study (Ericson et al., 2003; Goto et al., 2004). The finite helical axis method was used to calculate the DFA with a 30° interval and 5° increasing step (Woltring et al., 1985; Ericson et al., 2003; Goto et al., 2004), i.e., the screw axis calculated from 0° to 30° represented the DFA at 0°, while the next screw axis calculated from 5° to 35° represented the DFA at 5°. The TCA was defined as the fitted cylinder centerline on the surface of the humeral trochlear. The elbow motion was divided into four phases with a full-extension (FE)–30°, 30°–60°, 60°–90°, and 90°–maximal flexion (MF) (Figure 1C). The MFA was defined as taking the average position of all DFAs. The average DFA at each phase was calculated as the average of the screw axes in each phase, and its position was defined as the inclination with the MLA in the transverse and coronal planes. The range of DFA was also revealed at a different phase of motion in coronal and transverse views. The intercept point of DFA with the medial and lateral sagittal planes, perpendicular to TCA, illustrates its continual dynamic process of elbow flexion. The coronal plane was defined as the plane determined by the humeral long axis and MLA. The DFA intercept sagittal plane of each subject was normalized to the average articular width of 39.2 mm (from the most lateral circle on the capitellum to the most medial circle on the trochlea).

### Measurement of Elbow Morphological Parameters

The 3D bone models were imported into Amira (v.6.7.0, Thermo Fisher Scientific, Waltham, MA, United States) to measure the morphological parameters of the distal humerus, following the method in the article of Desai et al. (2014) in the anterior–posterior plane and the axis plane. In total, eight parameters (Figure 1D) were obtained, namely, the lateral trochlear height (LTH), trochlear height (TH), medial trochlear height (MTH), trochlear width (TW), trochlear width proper (TWP), lateral trochlear depth (LTD), trochlear groove depth (TD), and medial trochlear depth (MTD). Some parameters (LTH and LTD) were repeatedly measured by the same observer (DY-Z) and co-author (XJ-H) to test our reliability. The intraclass correlation coefficients (ICC) of inter-observer and intra-observer reliabilities were 0.98 and 0.96 for the LTH measurement and were 0.97, and 0.98 for the LTD measurement.

### Statistical Analysis

A *post hoc* power analysis was performed to estimate the statistical power (1-β), with a medium effect size and a = 0.05, using statistical power analysis software (G*Power version 3.1). The Mann–Whitney *U* test was performed to compare the difference in morphology parameters. The Spearman correlation analysis was used to calculate the relationship between distal humeral morphology parameters and DFA changes at different ranges. A statistical analysis was performed by numeral calculation software MATLAB (MATLAB, MathWorks, Natick, MA, United States). The significance level was set at 0.05 for all statistical analyses.

## Results

### Mean Flexion Axis and Trochlear Central Axis

**FIGURE 2:**
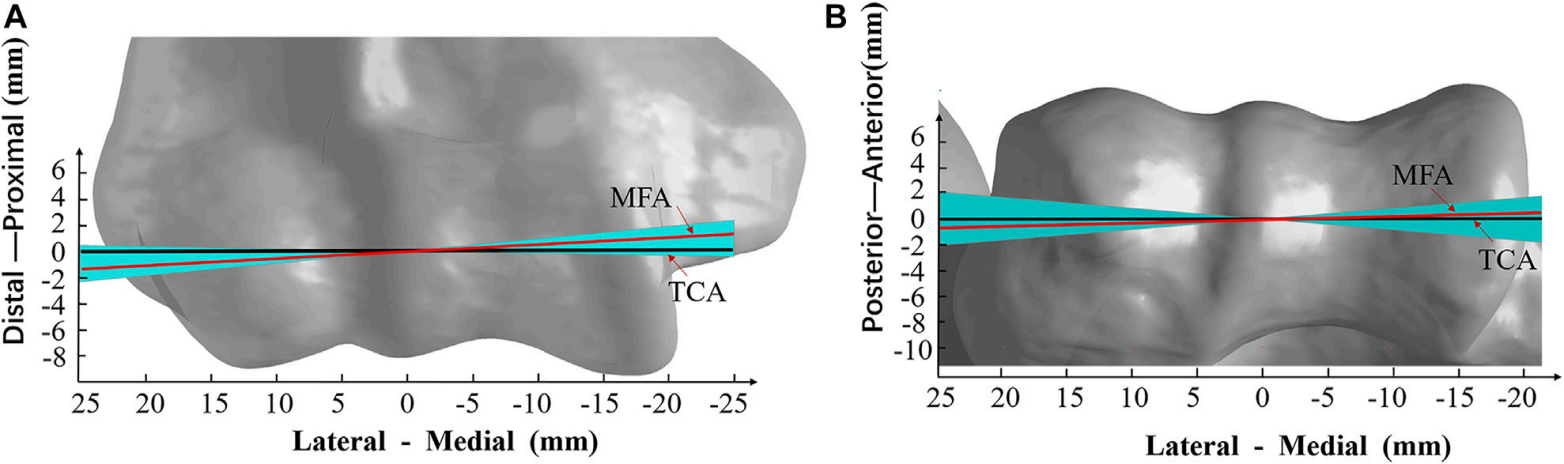
Varus–valgus and internal rotation–external rotation range of the mean flexion axis (MFA). **(A)** Varus–valgus (coronal plane view) and **(B)** internal rotation–external rotation (transverse plane view) of the MFA from all trials of the subject are shown. The solid red line is the mean position of MFA (3.1° varus and 1.3° internal rotation); the black line is the trochlea central axis (TCA). The light blue area represents the deviation of MFA within all subjects (5.4° varus maximum to 1.0° valgus minimum, and 4.8° external rotation to 4.7° internal rotation); the models of the humerus are all normalized to the average TCA length of 39.2 mm.

The average orientation of MFA was 3.1° ± 1.9° varus (range, 5.4° varus–1.0° valgus) relative to the TCA in the coronal plane among all the subjects (Figure 2A). The angle between the MFA and TCA was 1.3° ± 3.0° (range, 4.7° internal rotation–4.8° external rotation) in the transverse plane (Figure 2B). Also, the 3D spatial angle between MFA and TCA was 5.2° ± 1.4° (3.4°–7.3°).

**FIGURE 3:**
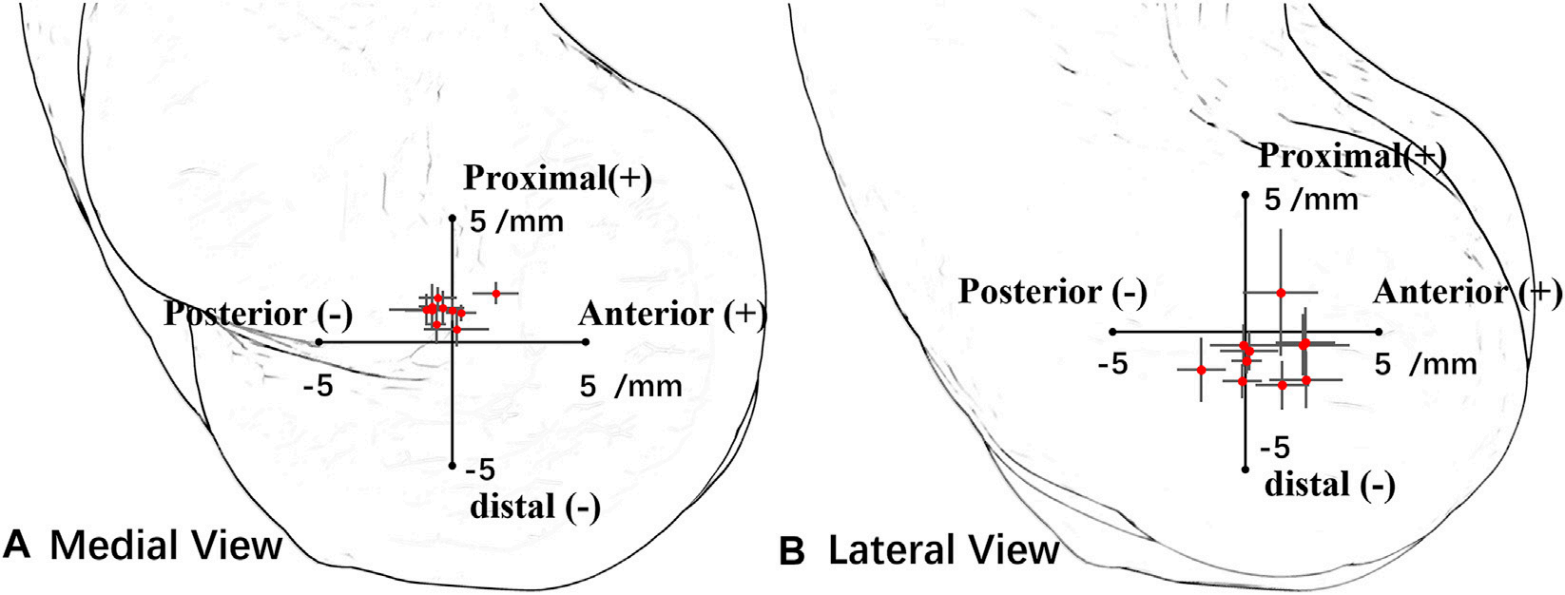
Position of all subjects’ mean flexion axis (MFA) intersection points in the lateral plane and medial plane. Red points represent the average center, and the horizontal and vertical lines represent the standard deviation of the axis in the anterior–posterior direction and the distal–proximal direction. **(A)** In the medial view, the origin point is the medial trochlear center. **(B)** In the lateral view, the origin point is the lateral trochlear center.

The MFA was located in 0.3 ± 0.3 mm (range −0.2 to 0.8 mm) anterior and 0.2 ± 0.4 mm (range −0.2 to 1.1 mm) distal related to the center of the trochlear on the medial sagittal plane (Figure 3A). The MFA was located in 0.8 ± 1.3 mm (range −1.7 to 2.3 mm) anterior and −0.8 ± 1.0 mm (range −1.9 to 1.4 mm) distal related to the lateral trochlear center on the lateral sagittal plane (Figure 3B; Table 1).

**TABLE 1:** Mean flexion axis (MFA) position in the coronal, transverse, lateral sagittal, and medial sagittal planes. Positive inclination indicated that MFA was varus and internally rotated to the medial–lateral epicondyle axis (MLA). In the lateral and medial planes, positive values indicated that the MFA was anterior and proximal to the center of the trochlea.

Subject	Coronal plane/°	Transverse plane/°	Lateral plane (mm)		Medial plane (mm)	
			Lateral X	Lateral Y	Medial X	Medial Y
1	−4.0 ± 1.5	0.0 ± 2.4	0.1 ± 1.1	−0.7 ± 0.7	0.3 ± 0.6	1.2 ± 0.3
2	1.6 ± 1.9	1.4 ± 2.4	1.4 ± 1.0	−1.9 ± 0.9	−0.5 ± 0.7	1.8 ± 0.5
3	−3.2 ± 3.1	−2.8 ± 2.2	2.3 ± 1.1	−0.4 ± 1.3	−0.8 ± 0.5	1.4 ± 0.9
4	−4.9 ± 1.7	3.1 ± 2.1	−0.1 ± 0.7	−1.8 ± 0.6	−0.4 ± 0.8	1.4 ± 0.7
5	−9.0 ± 4.4	0.8 ± 2.2	1.3 ± 1.4	1.4 ± 2.3	−0.6 ± 0.4	0.7 ± 0.8
6	−4.5 ± 2.3	−6.9 ± 2.3	−1.7 ± 0.9	−1.4 ± 1.2	1.6 ± 0.9	2.0 ± 0.5
7	1.8 ± 1.1	−2.8 ± 1.1	0.0 ± 0.6	−1.0 ± 0.5	0.0 ± 0.3	1.3 ± 0.4
8	−0.4 ± 1.4	3.4 ± 2.5	2.3 ± 1.4	−1.7 ± 1.0	−0.8 ± 1.0	1.3 ± 0.7
9	−6.6 ± 1.1	−1.3 ± 3.5	−0.1 ± 1.3	−0.5 ± 0.8	0.2 ± 1.2	0.5 ± 0.7
10	−9.0 ± 1.7	−0.8 ± 4.4	2.2 ± 1.8	−0.5 ± 1.1	−1.0 ± 1.4	1.3 ± 0.6

### Dynamic Flexion Axis of the Ulna Relative to the Humerus

**FIGURE 4:**
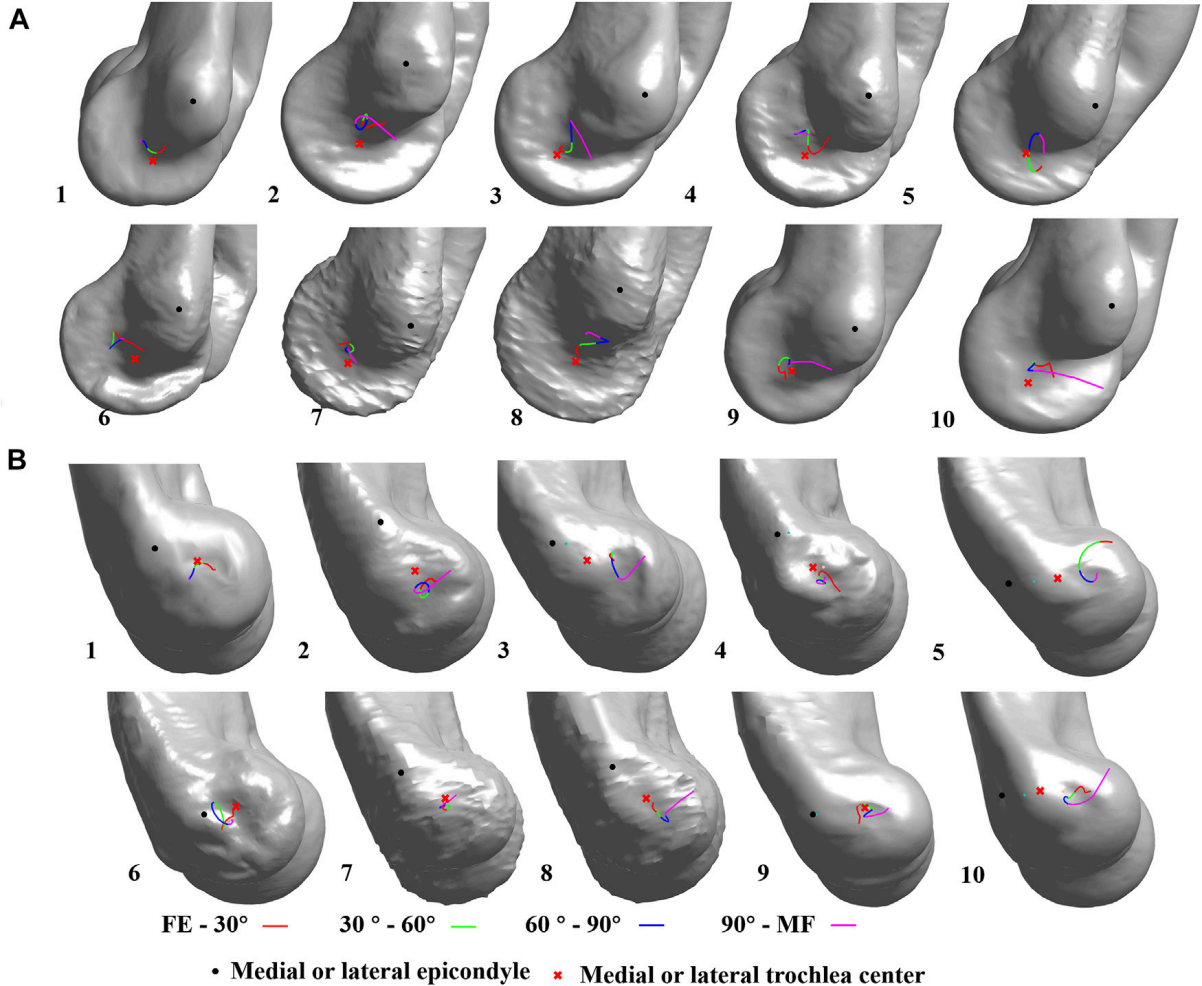
Dynamic flexion axis (DFA) change among all subjects. The continuous change in the DFA is represented by four colors which indicate four flexion–extension phases. FE, full-extension; MF, maximum flexion; **(A)** medial view; **(B)** lateral view.

The pathway of the intercept points of the DFA did not show a regular pattern during the flexion for all the subjects (Figure 4). On the lateral sagittal plane, the trajectory of the intercepts twined around the lateral trochlear center within an area of 4.4 mm ×  3.7 mm. On the medial sagittal plane, the trajectory of the intercepts moved above the medial trochlear center and was laid within an area of 3.1 mm ×  2.1 mm. The positions and range of an average DFA at the difference flexion phase are shown in Table 2. At the coronal plane, the average DFA showed the valgus to medial–lateral epicondyle axis at all flexion phases. At the transverse plane, the average DFA showed an external rotation relative to the medial–-lateral epicondyle axis except during the flexion phase from 90° to maximal flexion.

**TABLE 2:** Values representing the angle between the average rotation axis and medial–lateral epicondyle axis in the coronal plane and transverse plane at different phases of flexion. Positive values indicate the average rotation axis was varus and internally rotated to the medial–lateral epicondyle axis (MLA) (FE–30, full extension to 30° flexion; 30–60, 30° flexion to 60° flexion; 60–90, 60° flexion to 90° flexion; 90–max, 90° flexion to max flexion; FE–MF, full extension to max flexion).

	Coronal plane (°)				Transverse plane(°)			
	Mean/std	Max/std	Min/std	ROM/std	Mean/std	Max/std	Min/std	Range/std
FE–30	−5.2/5.1	1.2/3.8	−6.6/5	2.8/1.9	−0.8/3.5	3.2/4.1	−2.4/3.3	3.6/2.4
30–60	−4.2/4.8	−2.7/4.3	−5.7/5.3	3.0/2.3	−1.4/3.3	−0.2/3.7	−2.5/3.5	2.3/1.2
60–90	−3/3.5	−1.7/3.6	−4.3/3.7	2.6/2	−1.3/4.2	0/4.1	−2.5/4.0	2.5/1.3
90–MF	−2.9/3.6	−2.7/4.3	−5.8/4.3	4.1/3.0	1.0/3.5	−0.2/3.7	−1.4/3.2	6.0/5.6
FE–MF	−3.8/3.8	−1.2/3.8	−7.9/4.7	6.7/2.5	−0.6/3.1	−5.9/4.3	−4.0/2.8	9.9/4.1

### Distal Humeral Morphological Parameters

The sagittal diameters of the medial trochlear, lateral trochlear, and trochlear were 21.4 ± 1.8 mm, 19.0 ± 1.7 mm, and 15.2 ± 1.3 mm. The mean MTH (range 18.6–24.8 mm) was significantly (*p* = 0.0071) larger than LTH. The LTH (range 16.3–22.5 mm) was significantly (*p* < 0.0001) larger than TH (range 13.3–17.2 mm) amount subjects (MTH > LTH > TH). Likewise, the MTD was significantly larger than LTD (*p* = 0.0011), and LTD was significantly larger than TD (*p* < 0.0001) (MTD > LTD > TD). The depth values of MTD, LTD, and TD were 22.5 ± 1.7 mm, 19.8 ± 1.3 mm, and 16.2 ± 1.1 mm, respectively (Table 3).

**TABLE 3:** Result of all morphological parameters.

	MTD	TD	LTD	TWP	TW	MTH	TH	LTH
Average (mm)	22.5	16.2	19.8	18.4	22.8	21.4	15.2	19.0
STD (mm)	1.7	1.1	1.3	2.1	2.3	1.8	1.3	1.7
Max/min	25.7/19.7	17.5/14.5	22.0/17.3	20.9/13.5	25.5/18.0	24.8/18.6	17.2/13.3	22.5/16.3

### Correlation Between Morphological Parameters and DFA

The different morphological parameters were correlated with the positions and ranges of the DFA at different phases. From full extension to a 30° flexion, MTD (*r* = 0.68 and *p* = 0.03) and MTH (*r* = 0.69 and *p* = 0.03) were all positively correlated with the range of DFA (2.8° ± 1.9°) in the coronal plane. From flexion of 30° to 60°, LTH (*r* = −0.64 and *p* = 0.04) was negatively correlated with the position of average DFA (−1.4° ± 3.3°) in the transverse plane. LTH (*r* = 0.71 and *p* = 0.02) was positively correlated with the range of DFA (3.0 ± 2.3°) in the coronal plane. From flexion of 60° to 90°, a significant correlation was found between LTH with the position of average DFA in the coronal (*r* = −0.77 and *p* = 0.01) and transverse planes (*r* = −0.76 and *p* = 0.01). From a flexion of 90° to maximal flexion, TW (*r* = 0.66 and *p* = 0.04) and TWP (*r* = 0.67 and *p* = 0.04) were all positively correlated with the range of DFA (6.0° ± 5.6°) in the transverse plane. During the whole range of flexion motion, significant correlation was found among LTH with the position of the average DFA in the coronal (*r* = −0.64 and *p* = 0.04) and transverse planes (*r* = −0.71 and *p* = 0.01) (Table 4).

**TABLE 4:** Correlation between the morphological parameters with the position of average DFA and range of DFA in different phases of flexion (FE, full extension; MF, maximal flexion).

Variable	Descriptive data/mm		Range of the DFA in the coronal plane (FE–30°)		
	Means ± SD	Range	Means ± SD/°	*r*	*p*	Means ± SD/°	*r*	*p*
MTD	22.5 ± 1.7	19.7–25.7	2.8 ± 1.9	0.68	0.03	—	—	—
MTH	21.4 ± 1.8	18.6–24.8		0.69	0.03	—	—	—
			Position of the average DFA in the transverse plane (30°–60°)	Range of the DFA in the coronal plane (30°–60°)				
LTH	19.0 ± 1.7	16.3–22.5	−1.4 ± 3.3	−0.64	0.04	3.0 ± 2.3	0.71	0.02
			Position of the average DFA in the coronal plane (30°–60°)	Position of the average DFA in the transverse plane (30°–60°)				
LTH	19.0 ± 1.7	16.3–22.5	−3.0 ± 3.5	−0.77	0.01	−1.3 ± 4.2	−0.76	0.01
			Range of the DFA in the transverse plane (60°–90°)	—				
TW	22.8 ± 2.3	18.0–25.5	6.0 ± 5.6	−0.66	0.04	—	—	—
TWP	18.4 ± 2.1	13.5–20.9	6.0 ± 5.6	−0.67	0.04	—	—	—
			Position of the average DFA in the coronal plane (FE–MF)	Position of the average DFA in the transverse plane (FE–MF)				
LTH	19.0 ± 1.7	16.3–22.5	−3.8 ± 3.8	−0.64	0.04	−0.6 ± 3.1	−0.71	0.02

## Discussion

This study quantified the difference between the MFA and TCA, investigated the *in vivo* continuous position of the DFA of the healthy human elbow joint during extension to flexion, and evaluated its relationship with distal humeral morphological parameters. First, we found that the MFA showed a mean angle of 3.1° ± 1.9° varus and an external rotation of 1.3° ± 3.0° relative to TCA. Also, MFA was closer to the trochlear center on the medial side but more away from the trochlear center on the lateral side. Second, the pathway of the DFA presented individual patterns. Finally, the widths of the lateral and medial trochlea were strongly correlated with the position of the DFA, while the size of the trochlear grove was not. These results indicated that the lateral and medial sizes of the trochlea were the key parameters that may affect the elbow joint function.

The position of MFA was varus and externally rotated to the TCA in the coronal and transverse planes, suggesting that the TCA may not be suitable to represent the elbow flexion axis. *In vivo* (Ericson et al., 2003; Ericson et al., 2008) and *in vitro* (Bottlang et al., 2000; Duck et al., 2003a) studies about the optimum elbow flexion axis have also been published. Bottlang et al. (2000) reported the range of MFA was 2.6° ± 1.0° in the coronal plane and 5.7° ± 2.2° in the transverse plane in a passive movement by using the electromagnetic motion tracking method. Ericson et al. (2003) found greater variations in the coronal plane (6.2° varus to 6.5° valgus) than in the transverse plane (2.4 ° internal rotation to 2.2 ° external rotation) in an *in vivo* active elbow movement situation. Those variations were close to our result (coronal plane: 5.4° varus to 1.0° valgus, transverse plane: 4.7° internal rotation to 4.8° external rotation). Bottlang et al. (2000) reported that the DFA intersection has the smallest distribution near the medial trochlea, located 2.0 ± 0.6 mm away from the longitudinal axis of the humerus in the frontal plane. Goto et al. (2004) reported that the DFA intersection distribution tended to be scattered on the lateral trochlea than on the medial trochlea. Our study quantified the MFA intersection located on the medial trochlea (0.3 ± 0.3 mm anterior and 0.2 ± 0.4 mm distal) and the lateral trochlea (0.8 ± 1.3 mm anterior and −0.8 ± 1.0 mm distal), which showed a similar pattern that the distribution was more scattered on the lateral side and more concentrated on the medial side (Figure 3). The previous study has shown that the MCL provided a primary stabilizer to resist valgus stress and constrained the internal rotation of the forearm at the elbow (Labott et al., 2018). These results may explain the constrained function of the MCL on the medial side of the elbow.

Accurately aligning the implant or external fixation with the elbow is still a challenge for surgeons. Although the TCA is easy to recognize through the humeral trochlear feature, there is still a high alignment error. According to Wiggers et al. (2014), the mean errors of identifying the TCA were 8° of the mean rotation and 2 mm of the mean translation with a large inter-observer variability. The humeral stem loading will increase after humeral component mal-alignment, according to an *in vitro* study (Brownhill et al., 2012). For extra fixation mal-alignment conditions, increasing stresses might be transferred to the fixator pins and the pin–bone interface. They were potential factors that lead to clinical issues of pin loosening, pin breaking, or persistent instability (Stavlas et al., 2007). A more accurate alignment can be obtained through the computer navigation technology. McDonald et al. (2010) improved the implant alignment errors to below 2° and 2 mm in total elbow arthroplasty, according to the reference of TCA by applying the image-based navigation system. However, the MFA could represent the elbow flexion better than the TCA. An accurate alignment of TCA could still cause abnormal stress at the implant–bone interface during flexion. According to our result, the MFAs showed individual positions, which complicated the alignment of the MFA. Surgeons could get an individual and accurate MFA alignment by combining the MFA calculation algorithm and computer navigation technology (Song et al., 2018).

Previous studies have reported that the DFA pathway is a roller configuration during elbow flexion (Bottlang et al., 2000; Goto et al., 2004). Those studies using electromagnetic tracking and passive or simulative active cadaveric elbow might reproduce high repeatability of regular movement, which leads to a regular pattern of the axis. The only study reported by Ericson et al. (2003) revealed an irregular DFA pattern during *in vivo* elbow flexion, which was similar to our finding. The variation in the subjects’ muscle activity, joint laxity, and geometry may influence the movement that results in an individual pattern of DFA and differ from those *in vitro* studies. It has been proven that the DFA movement pattern would be affected by various factors such as ligament, forearm position, or muscle force (Duck et al., 2003a; Duck et al., 2003b). Duck et al. (2003a) reported that a division of an elbow ligament led to the deviation of DFA displacement and active movement of the elbow by simulating the muscle force provided more stability of the axis than passive motion. Duck et al. (2003b) also reported that the mean DFA was more externally rotated by 1° with the forearm held supinated rather than pronated. These results may suggest the movement pattern of DFA may reflect the biomechanical condition of the elbow.

Geometry variation, as one of the biomechanical factors, should be considered in the TEA implant design and treatment of distal humerus fracture (Shiba et al., 1988; Desai et al., 2014; Lapner et al., 2014). Few studies had analyzed the distal humerus morphologic sizes based on CT/MRI models or cadavers (Desai et al., 2014; Giannicola et al., 2017). Desai et al. (2014) showed that the mean MTH and LTD were 29.9 and 21.6 mm, which are both significantly larger than the TH’s 17.8 mm. Our study revealed that the distal humeral articular surface had a concave barrel-shaped trochlea with a circular concavity and showed similar sizes of the trochlea with MTH, LTH, and TH which were 21.4, 19.0, and 15.2 mm, respectively. A new kind of an implant that followed this relationship was proved to have significantly better ulnohumeral contact (Lapner et al., 2014; Willing et al., 2014). It might reduce the contact pressure and increase survival time. However, Kamineni et al. (2005) suggested that the implant design, which does not precisely match the elbow, does not significantly affect the elbow function. Thus, the information about the geometry and the function of the elbow requires further investigation.

Some other research had evaluated the effects of the anatomical variation on the biomechanical changes of the joint. Lenoir et al. (2015b) revealed that the anterior angulation of the humerus led to a humeral component lateral offset, which was associated with pain intensity. Some studies revealed that the articular geometry changes reflected the knee kinematics and the moment arm (Bull et al., 2008; Gray et al., 2021). Our study showed a strong correlation between humeral trochlear morphological features and the position of the DFA in the different phases of flexion. 1) At the early stage of flexion, our study found the shape of the medial trochlear, which means that MTH and MTD were correlated with the DFA range from full extension to 30° flexion. Goto et al. (2004) reported that the contact area of the humeroulnar joint was mainly concentrated on the medial trochlea during early flexion. When the height or depth of the medial trochlea increases, it may cause a change in the joint contact pattern, which might explain the range of the DFA change at the early flexion phase. 2) During the phase of flexion, from 30° to 90° of flexion, the LTH was considered an important feature that would affect the position of the average DFA in the transverse or coronal planes. Giannicola et al. (2016) found that there were significant individual differences in the trochlear notch angle. The increasing size of the lateral side of the trochlea would directly decrease the angle of the trochlear notch, thereby causing the inherent position of the humeroulnar joint to change, leading to changes in the DFA position. 3) During the phase of flexion from 90° to the maximum flexion angle, the TW and the TWP were the essential factors that would affect the range of the DFA. 4) Our study also found that TH or TD did not correlate with the DFA position or range at all phases of flexion. According to the research by Lapner et al. (2014), when the anatomical shaping humeral prosthesis was applied to the specimen, a significant edge wearing was visited from the humeral spool on the proximal ulna surface after simulation testing. At the same time, the trochlear groove did not show noticeable wearing on the ulna. It was also evident that the feature of the trochlear groove was not as essential as the size of the medial or lateral trochlea. Therefore, this information implies that doctors or engineers when repairing the articular surface of the humerus or designing the distal humerus prosthesis, should pay more attention as the medial and lateral sizes of the trochlea were important characteristic factors.

The present study should be interpreted in light of its potential limitations. First, we only recruited 10 subjects, so it was impossible to compare the effects of gender, age, and other factors on the rotation axis of the elbow joint. The result of the *post hoc* power analysis (0.97, which is higher than 0.8) proved that the sample size was sufficient to reflect statistical differences. Second, all the subjects were healthy and could not evaluate the relationship between the shape of the trochlea and the DFA after the elbow joint’s repair. Further studies of cadaveric specimens might be used to verify our findings.

## Conclusion

In summary, the current study investigated the relationship between the *in vivo* elbow dynamic flexion axis location and the distal humeral trochlear geometry. The inclination of MFA was found to be 3.1° varus and 1.3° external rotation to the TCA. Significant inter-individual differences in the pattern of the dynamic *in vivo* DFA were observed in our study. The lateral and medial sizes of the trochlea were significantly correlated with the position and range of the DFA, which indicated that they were the key parameters that might affect the elbow joint flexion function. When recovering complex distal humeral fractures or considering the TEA implant design, surgeons should pay more attention to the lateral and medial trochlear geometry.

## Data Availability

DZ and XH made substantial contributions to the conception and design, acquisition and analysis, and interpretation of the data, were involved in drafting the article, and gave final approval of the version to be published. K-NA and KD each partially helped in the article revision, and all gave final approval of the version to be published. XY provided some suggestions and helped in article revise during article review time. WG and T-YT made contributions to the conception and design, were involved in revising it critically for important intellectual content, and gave final approval of the version to be published. All authors contributed to the article and approved the submitted version.

## References

[B1] Bottlang M. Madey S. M. Steyers C. M. Marsh J. L. Brown T. D. (2000). Assessment of Elbow Joint Kinematics in Passive Motion by Electromagnetic Motion Tracking. J. Orthop. Res. 18 (2), 195–202. 10.1002/jor.1100180206 10815819

[B2] Brownhill J. R. Furukawa K. Faber K. J. Johnson J. A. King G. J. W. (2006). Surgeon Accuracy in the Selection of the Flexion-Extension axis of the Elbow: an *In Vitro* Study. J. Shoulder Elb. Surg. 15 (4), 451–456. 10.1016/j.jse.2005.09.011 16831650

[B3] Brownhill J. R. King G. J. W. Johnson J. A. (2007). Morphologic Analysis of the Distal Humerus with Special Interest in Elbow Implant Sizing and Alignment. J. Shoulder Elb. Surg. 16 (3 Suppl. l), S126–S132. 10.1016/j.jse.2006.01.018 17408979

[B4] Brownhill J. R. Pollock J. W. Ferreira L. M. Johnson J. A. King G. J. W. (2012). The Effect of Implant Malalignment on Joint Loading in Total Elbow Arthroplasty: an *In Vitro* Study. J. Shoulder Elb. Surg. 21 (8), 1032–1038. 10.1016/j.jse.2011.05.024 21868256

[B5] Bull A. M. J. Kessler O. Alam M. Amis A. A. (2008). Changes in Knee Kinematics Reflect the Articular Geometry after Arthroplasty. Clin. Orthop. Relat. Res. 466 (10), 2491–2499. 10.1007/s11999-008-0440-z 18704612 PMC2584306

[B6] Desai S. J. Deluce S. Johnson J. A. Ferreira L. M. Leclerc A. E. Athwal G. S. (2014). An Anthropometric Study of the Distal Humerus. J. Shoulder Elb. Surg. 23 (4), 463–469. 10.1016/j.jse.2013.11.026 24560468

[B7] Wiggers J. K. Dobbe J. G. G. Streekstra G. J. Schep N. W. L. Snijders R. M. den Hartog D. (2017). Accuracy in Identifying the Elbow Rotation axis on Simulated Fluoroscopic Images Using a New Anatomical Landmark. Strateg. Trauma Limb Reconstr. 12 (3), 133–139. 10.1007/s11751-017-0289-3 PMC5653598 28593358

[B8] Dos Santos A. Creze M. Begin M. Laemmel E. Assabah B. Soubeyrand M. (2017). Cadaveric Assessment of a 3D-Printed Aiming Device for Implantation of a Hinged Elbow External Fixator. Eur. J. Orthop. Surg. Traumatol. 27 (3), 405–414. 10.1007/s00590-016-1889-1 27942933

[B9] Duck T. R. Dunning C. E. Armstrong A. D. Johnson J. A. King G. J. W. (2003a). Application of Screw Displacement Axes to Quantify Elbow Instability. Clin. Biomech. 18 (4), 303–310. 10.1016/s0268-0033(03)00021-4 12689780

[B10] Duck T. R. Dunning C. E. King G. J. W. Johnson J. A. (2003b). Variability and Repeatability of the Flexion axis at the Ulnohumeral Joint. J. Orthop. Res. 21 (3), 399–404. 10.1016/s0736-0266(02)00198-5 12706011

[B11] Ehrig R. M. Heller M. O. (2019). On Intrinsic Equivalences of the Finite Helical axis, the Instantaneous Helical axis, and the SARA Approach. A Mathematical Perspective. J. Biomechanics 84, 4–10. 10.1016/j.jbiomech.2018.12.034 30661733

[B12] Ehrig R. M. Taylor W. R. Duda G. N. Heller M. O. (2007). A Survey of Formal Methods for Determining Functional Joint Axes. J. Biomechanics 40 (10), 2150–2157. 10.1016/j.jbiomech.2006.10.026 17169365

[B13] Ericson A. Arndt A. Stark A. Wretenberg P. Lundberg A. (2003). Variation in the Position and Orientation of the Elbow Flexion axis. J. Bone Jt. Surg. Br. volumeBritish volume 85-B (4), 538–544. 10.1302/0301-620x.85b4.13925 12793560

[B14] Ericson A. Stark A. Arndt A. (2008). Variation in the Position of the Elbow Flexion axis after Total Joint Replacement with Three Different Prostheses. J. Shoulder Elb. Surg. 17 (5), 760–767. 10.1016/j.jse.2008.03.003 18619867

[B15] Geurts E. J. Viveen J. van Riet R. P. Kodde I. F. Eygendaal D. (2019). Outcomes after Revision Total Elbow Arthroplasty: a Systematic Review. J. Shoulder Elb. Surg. 28 (2), 381–386. 10.1016/j.jse.2018.08.024 30658776

[B16] Giannicola G. Scacchi M. Sedati P. Gumina S. (2016). Anatomical Variations of the Trochlear Notch Angle: MRI Analysis of 78 Elbows. Musculoskelet. Surg. 100 (Suppl. 1), 89–95. 10.1007/s12306-016-0407-2 27900706

[B17] Giannicola G. Spinello P. Scacchi M. Gumina S. (2017). Cartilage Thickness of Distal Humerus and its Relationships with Bone Dimensions: Magnetic Resonance Imaging Bilateral Study in Healthy Elbows. J. Shoulder Elb. Surg. 26 (5), e128–e136. 10.1016/j.jse.2016.10.012 28131685

[B18] Gordon B. J. Dapena J. (2013). A Method to Determine the Orientation of the Upper Arm about its Longitudinal axis during Dynamic Motions. J. Biomechanics 46 (1), 97–101. 10.1016/j.jbiomech.2012.10.011 23141956

[B19] Goto A. Moritomo H. Murase T. Oka K. Sugamoto K. Arimura T. (2004). *In Vivo* elbow Biomechanical Analysis during Flexion: Three-Dimensional Motion Analysis Using Magnetic Resonance Imaging. J. Shoulder Elb. Surg. 13 (4), 441–447. 10.1016/j.jse.2004.01.022 15220886

[B20] Gray H. A. Guan S. Thomeer L. T. Pandy M. G. (2021). Moment Arm of the Knee-Extensor Mechanism Measured *In Vivo* across a Range of Daily Activities. J. Biomechanics 123, 110484. 10.1016/j.jbiomech.2021.110484 34062347

[B21] Hua K. Ji S. Li T. Chen C. Zha Y. Gong M. (2020). Correlation between Modified Trochleocapitellar Index and Post-traumatic Elbow Stiffness in Type C2-3 Distal Humeral Fractures Among Adults. J. Shoulder Elb. Surg. 29 (9), 1876–1883. 10.1016/j.jse.2020.02.016 32446760

[B22] Kamineni S. OʼDriscoll S. W. Urban M. Garg A. Berglund L. J. Morrey B. F. (2005). Intrinsic Constraint of Unlinked Total Elbow Replacements-The Ulnotrochlear Joint. J. Bone & Jt. Surg. 87 (9), 2019–2027. 10.2106/jbjs.C.00983 16140818

[B23] Krukhaug Y. Hallan G. Dybvik E. Lie S. A. Furnes O. N. (2018). A Survivorship Study of 838 Total Elbow Replacements: a Report from the Norwegian Arthroplasty Register 1994-2016. J. Shoulder Elb. Surg. 27 (2), 260–269. 10.1016/j.jse.2017.10.018 29332662

[B24] Labott J. R. Aibinder W. R. Dines J. S. Camp C. L. (2018). Understanding the Medial Ulnar Collateral Ligament of the Elbow: Review of Native Ligament Anatomy and Function. Wjo 9 (6), 78–84. 10.5312/wjo.v9.i6.78 29984194 PMC6033709

[B25] Lapner M. Willing R. Johnson J. A. King G. J. W. (2014). The Effect of Distal Humeral Hemiarthroplasty on Articular Contact of the Elbow. Clin. Biomech. 29 (5), 537–544. 10.1016/j.clinbiomech.2014.03.010 24780463

[B26] Lenoir H. Chammas M. Micallef J. P. Lazerges C. Waitzenegger T. Coulet B. (2015a). The Effect of the Anatomy of the Distal Humerus and Proximal Ulna on the Positioning of the Components in Total Elbow Arthroplasty. Bone & Jt. J. 97-b (11), 1539–1545. 10.1302/0301-620x.97b11.36071 26530658

[B27] Lenoir H. Micallef J. P. Djerbi I. Waitzenegger T. Lazerges C. Chammas M. (2015b). Total Elbow Arthroplasty: Influence of Implant Positioning on Functional Outcomes. Orthop. Traumatology Surg. Res. 101 (6), 721–727. 10.1016/j.otsr.2015.07.008 26372184

[B28] Mansat P. Bonnevialle N. Rongières M. Mansat M. Bonnevialle P. (2013a). Results with a Minimum of 10 Years Follow-Up of the Coonrad/Morrey Total Elbow Arthroplasty. Orthop. Traumatology Surg. Res. 99 (6), S337–S343. 10.1016/j.otsr.2013.07.002 23932914

[B29] Mansat P. Nouaille Degorce H. Bonnevialle N. Demezon H. Fabre T. (2013b). Total Elbow Arthroplasty for Acute Distal Humeral Fractures in Patients over 65 Years Old - Results of a Multicenter Study in 87 Patients. Orthop. Traumatology Surg. Res. 99 (7), 779–784. 10.1016/j.otsr.2013.08.003 24095596

[B30] McDonald C. P. Johnson J. A. Peters T. M. King G. J. W. (2010). Image-based Navigation Improves the Positioning of the Humeral Component in Total Elbow Arthroplasty. J. Shoulder Elb. Surg. 19 (4), 533–543. 10.1016/j.jse.2009.10.010 20137975

[B31] McDonald C. P. Moutzouros V. Bey M. J. (2012). Measuring Dynamic *In-Vivo* Elbow Kinematics: Description of Technique and Estimation of Accuracy. J. Biomech. Eng. 134 (12), 124502. 10.1115/1.4007951 23363209

[B32] Muriuki M. G. Mohagheh-Motlagh A. Smolinski P. J. Miller M. C. (2012). Elbow Helical Axes of Motion Are Not the Same in Physiologic and Kinetic Joint Simulators. J. Biomechanics 45 (13), 2289–2292. 10.1016/j.jbiomech.2012.06.021 22784652

[B33] Pham T. T. Delclaux S. Huguet S. Wargny M. Bonnevialle N. Mansat P. (2018). Coonrad-Morrey Total Elbow Arthroplasty for Patients with Rheumatoid Arthritis: 54 Prostheses Reviewed at 7 years' Average Follow-Up (Maximum, 16 years). J. Shoulder Elb. Surg. 27 (3), 398–403. 10.1016/j.jse.2017.11.007 29306664

[B34] Sanchez-Sotelo J. (2011). Total Elbow Arthroplasty. Toorthj 5, 115–123. 10.2174/1874325001105010115 PMC3093740 21584200

[B35] Schoch B. Wong J. Abboud J. Lazarus M. Getz C. Ramsey M. (2017). Results of Total Elbow Arthroplasty in Patients Less Than 50 Years Old. J. Hand Surg. 42 (10), 797–802. 10.1016/j.jhsa.2017.06.101 28823536

[B36] Shiba R. Sorbie C. Siu D. W. Bryant J. T. Cooke T. D. V. Wevers H. W. (1988). Geometry of the Humeroulnar Joint. J. Orthop. Res. 6 (6), 897–906. 10.1002/jor.1100060614 3171770

[B37] Song J. Ding H. Han W. Wang J. Wang G. (2018). An X-ray-free Method to Accurately Identify the Elbow Flexion-Extension axis for the Placement of a Hinged External Fixator. Int. J. CARS 13 (3), 375–387. 10.1007/s11548-017-1680-8 29101641

[B38] Stavlas P. Jensen S. L. Søjbjerg J. O. (2007). Kinematics of the Ligamentous Unstable Elbow Joint after Application of a Hinged External Fixation Device: a Cadaveric Study. J. Shoulder Elb. Surg. 16 (4), 491–496. 10.1016/j.jse.2006.07.012 17321152

[B39] Stokdijk M. Meskers C. G. M. Veeger H. E. J. de Boer Y. A. Rozing P. M. (1999). Determination of the Optimal Elbow axis for Evaluation of Placement of Prostheses. Clin. Biomech. 14 (3), 177–184. 10.1016/s0268-0033(98)00057-6 10619105

[B40] Wiggers J. K. Streekstra G. J. Kloen P. Mader K. Goslings J. C. Schep N. W. L. (2014). Surgical Accuracy in Identifying the Elbow Rotation axis on Fluoroscopic Images. J. Hand Surg. 39 (6), 1141–1145. 10.1016/j.jhsa.2014.03.008 24785699

[B41] Willing R. Lapner M. King G. J. W. Johnson J. A. (2014). *In Vitro* assessment of the Contact Mechanics of Reverse-Engineered Distal Humeral Hemiarthroplasty Prostheses. Clin. Biomech. 29 (9), 990–996. 10.1016/j.clinbiomech.2014.08.015 25238687

[B42] Woltring H. J. Huiskes R. de Lange A. Veldpaus F. E. (1985). Finite Centroid and Helical axis Estimation from Noisy Landmark Measurements in the Study of Human Joint Kinematics. J. Biomechanics 18 (5), 379–389. 10.1016/0021-9290(85)90293-3 4008508

[B43] Wu G. van der Helm F. C. T. Veeger H. E. J. Makhsous M. Van Roy P. Anglin C. (2005). ISB Recommendation on Definitions of Joint Coordinate Systems of Various Joints for the Reporting of Human Joint Motion-Part II: Shoulder, Elbow, Wrist and Hand. J. Biomechanics 38 (5), 981–992. 10.1016/j.jbiomech.2004.05.042 15844264

